# Clinical correlates of thalamus volume deficits in anti-psychotic-naïve schizophrenia patients: A 3-Tesla MRI study

**DOI:** 10.4103/0019-5545.70975

**Published:** 2010

**Authors:** Naren P. Rao, Sunil Kalmady, Rashmi Arasappa, Ganesan Venkatasubramanian

**Affiliations:** Department of Psychiatry, National Institute of Mental Health and Neurosciences (NIMHANS), Bangalore - 560 029, India

**Keywords:** Schizophrenia, thalamus, morphometry, MRI, Positive symptoms, negative symptoms

## Abstract

**Background:**

Thalamus, the sensory and motor gateway to the cortex, plays an important role in cognitive and perceptual disturbances in schizophrenia. Studies examining the volume of the thalamus in schizophrenia have reported conflicting findings due to the presence of potential confounding factors such as low-resolution imaging and anti-psychotics. The thalamus volume in anti-psychotic-naïve patients determined using high-resolution 3-Tesla magnetic resonance imaging (MRI) has not yet been examined.

**Materials and Methods:**

Using 3-Tesla MRI, this study for the first time examined anti-psychotic-naïve schizophrenia patients (n=18; M:F:11:7) in comparison with healthy controls (n=19;M:F:9:10) group-matched for age, sex, handedness, education, and socioeconomic status. The volume of the thalamus was measured using a three-dimensional, interactive, semi-automated analysis with good inter-rater and intra-rater reliability. Psychopathology was assessed using the Scale for Assessment of Negative Symptoms (SANS) and the Scale for Assessment of Positive Symptoms (SAPS).

**Results:**

Right, left, and total thalamus volumes of patients were significantly smaller than those of controls after controlling for the potential confounding effect of intracranial volume. Thalamus volumes had significant positive correlation with positive symptoms score (SAPS) and significant negative correlation with negative symptoms score (SANS).

**Conclusions:**

Thalamus volume deficits in anti-psychotic-naïve schizophrenia patients support a neurodevelopmental pathogenesis. The contrasting correlation of thalamus volume deficits with psychopathology scores suggests that contrasting pruning aberrations underlie symptom genesis in schizophrenia.

## INTRODUCTION

**Figure 1:**
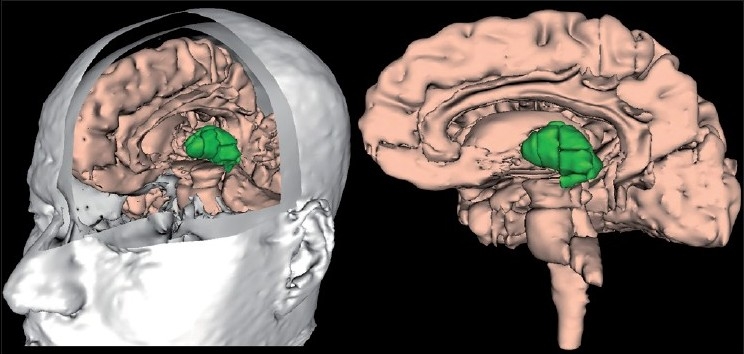
Anatomical location of Thalamus (depicted in green colour) in brain

Schizophrenia is proposed as a neurodevelopmental disorder involving dysfunctional cortical-subcortical circuitry.[1,2] The thalamus, a highly evolved sensory and motor gateway to the cortex, provides a nodal link between the cortex and the subcortical structures through cortico-striato-thalamic circuits.[3,4] The sensory and motor inputs are filtered and relayed by multiple thalamic nuclei to the cerebral cortex through cortico-striato-thalamic loops. In addition, the thalamus, in concert with the basal ganglia, also modulates the activity of the frontal cortex and is important in cognition.[4] Thus, the thalamus, a vital relay station to the cortex, is important for consciousness and perception as well as for the integration of thought processes.[1] For this function, the thalamus is positioned in a crucial anatomic location in the brain - rostrally within the diencephalon - i.e., it is interposed functionally and anatomically between the brainstem and the telencephalon [Figure 1].

Interestingly, impairment of these circuits is proposed to play an important role in the development of perceptual and cognitive disturbances in schizophrenia; aberration in thalamic functioning is suggested to contribute both to the perceptual disturbances[5,6] and the cognitive dysfunction (hypofrontality) seen in schizophrenia.[3,7]

Earlier, postmortem studies have documented morphological changes like cell loss and reduced tissue volume in the thalami of patients with schizophrenia.[8,9] Also, PET studies have shown abnormal activation of the thalami in patients with schizophrenia.[10,11] In addition, infarctions of the thalamus are associated with schizophrenia-like symptoms.[12,13] Several studies using functional imaging have found decreased thalamic activation in subjects with schizophrenia under a variety of test conditions, including tests of pre-pulse inhibition, working memory, selective attention, novelty processing, and verbal learning.[3,14,15]

Magnetic resonance imaging (MRI) has the advantage that it enables the study of the thalamus *in vivo* and avoids the potential confounders associated with postmortem studies. Nonetheless, MRI studies have given conflicting results regarding structural differences in the thalamus in schizophrenia. Although most studies have reported a relatively smaller volume of the thalamus in schizophrenia as compared to normal subjects, a substantial number of studies have found no difference in size between these two groups.[13,16] A meta-analysis has shown decreased size of the thalamus in patients with schizophrenia as compared to controls.[17] Recent reviews, in addition to confirming the earlier finding of smaller thalamic volume, reported multiple factors that could confound the results and possibly be the reason for the inconsistent findings.[13] Thus, these inconsistencies in the findings might have been secondary to differences in subject characteristics, for example, sex, handedness, age, and variations in symptom profile; in addition, the thalamus volume measurement methods and nonconsideration of total brain volume may also have contributed.[13] Use of low-resolution MRI to acquire images can also confound the results due to poor image quality - this is crucial, especially for the thalamus, since the gray-white delineation is suboptimal for reliable volumetric analysis, particularly in lesser-magnetic-strength imaging (for example, 1.5-Tesla). Studies examining the thalamus volume have used MRI of strength 0.5-1.5 Tesla; it is preferable to use a MRI of higher strength for better resolution and image quality.

Another important confounding factor is the effect of anti-psychotic medications, as psychotropic medications can have a neuroprotective role in schizophrenia by either preventing neuronal loss or by protecting against the pathophysiologic effect of the disease process.[18] In particular, both typical and atypical anti-psychotic medication can produce variations in thalamic volume. Dosage of the typical anti-psychotics was found to be significantly and positively correlated with total thalamic volume,[19,20] and atypical anti-psychotics were associated with significant expansion of the thalamus.[21,22] Examining drug-naïve patients is important to control for these confounding factors, but only a few studies have investigated drug-naïve patients.[23]

Though studies have shown a reduction in thalamus volume in schizophrenia, the relationship between thalamus volume deficits and psychopathology has been examined in very few studies and is yet to be established. Given its critical role in the neural network that is hypothesized to underlie schizophrenia pathogenesis, one would expect a significant association between thalamus volume deficit and psychopathology. Earlier studies have documented the association of negative symptoms with reduced gray matter volume in the prefrontal cortex and other brain areas;[24–27] interestingly, the thalamus is connected with most of these brain regions. Hence, one would expect a decrease in the volume of the thalamus to be associated with negative symptoms of schizophrenia.

In contrast, it has been hypothesized that the positive symptoms in schizophrenia may be associated with hyperconnectivity of certain brain regions[28] - for example, the frontal and temporal cortices. Indeed, a recent diffusion tensor imaging study has shown that fronto–temporal hyperconnectivity underlies the genesis of positive symptoms in schizophrenia.[29] This aberrant hyperconnectivity is also hypothesized to be of neurodevelopmental origin. Hence, while ‘aberrant pruning’ is associated with negative symptoms, ‘pathological blooming’ is hypothesized to be associated with positive symptoms.[28] As the thalamus is connected with these cortical regions, one would expect a larger thalamus volume to be associated with positive symptoms of schizophrenia.

In this study, using high-resolution 3-Tesla MRI for the first time, we examined the right thalamus volume, the left thalamus volume, and the total thalamic volume in anti-psychotic-naïve schizophrenia patients (*n*=18) in comparison with healthy controls (*n*=19) group-matched for age, sex, education, handedness, and socioeconomic status. In addition, we examined for a possible relation between symptom dimensions and thalamus volumes. Based on earlier reports (discussed above), we hypothesized that schizophrenia patients would have smaller thalamus volumes when compared to controls and predicted a contrasting correlation between positive and negative symptoms and thalamus volumes.

## MATERIAL AND METHODS

### Subjects

The study sample consisted of 18 drug-naïve schizophrenia patients and 19 healthy controls. Patients meeting the DSM-IV diagnostic criteria for schizophrenia were recruited from the outpatient department of National Institute of Mental Health and Neurosciences (NIMHANS), Bangalore, India. The diagnosis was established by applying Mini International Neuropsychiatric Interview-Plus (MINI-Plus).[30] An experienced psychiatrist confirmed the diagnosis through an independent clinical interview. Patients had no history of comorbid medical illness or any other psychiatric illness, including substance dependence. None of them were exposed to any kind of anti-psychotics or electroconvulsive therapy earlier or during the period of assessments. We collected relevant clinical information pertinent to illness onset and ascertained the anti-psychotic-naïve status via information obtained from the patient and family members. We assessed psychopathology using the Scale for the Assessment of Positive Symptoms (SAPS)[31] and the Scale for the Assessment of Negative Symptoms (SANS).[32]

Age, sex, and education-matched healthy controls who volunteered for participation in the study, were recruited by word of mouth. None of them had any psychiatric disorder as confirmed by MINI-Plus.[30] They had no history of medical illness or substance dependence. There was no family history of psychiatric illness, including alcohol dependence syndrome in first-degree relatives. We assessed handedness using Annett’s questionnaire;[33] all subjects were right-handed. Written informed consent was taken from all subjects before assessment. The study was approved by the NIMHANS ethics committee.

### Scanning protocol

MRI was done with a 3.0-Tesla scanner. T1-weighted three-dimensional magnetization-prepared rapid-acquisition gradient-echo sequence was performed (TR=8.1 msec, TE=3.7 msec, nutation angle=8°, FOV=256 mm, slice thickness 1 mm without interslice gap, NEX=1, matrix=256×256), yielding 165 sagittal slices. The images were stored with coded identification for blinded rating.

### Thalamus volume measurement

**Figure 2:**
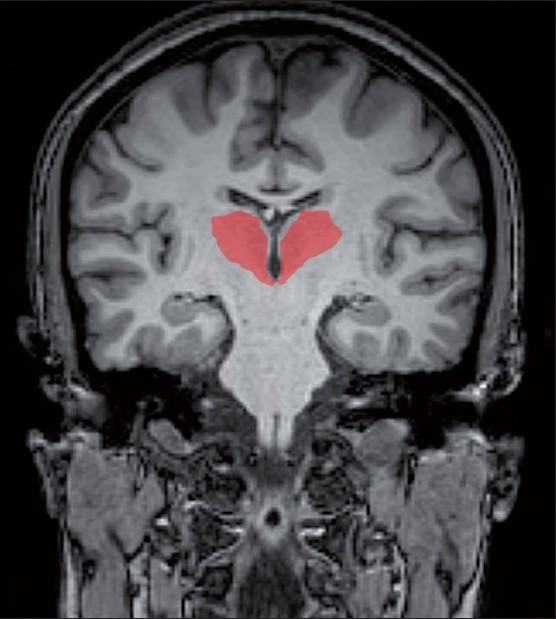
Thalamus (marked in red) in coronal section

We measured the right and left thalamus volumes and the total intracranial volume (which was used as a covariate). To facilitate delineation of the thalamus, the tracing was performed in segmented gray matter images. Neuroanatomical boundaries were determined by reference to standard neuroanatomical atlases, and detailed definitions were adapted from previously published psychiatric neuroimaging studies of the thalamus[23,34,35] [Figure 2]. We measured the region of interest (ROI) – the thalamus –using Medical Image Processing and Visualization (MIPAV) software. This software is in the public domain and can be downloaded from the internet (http://mipav.cit.nih.gov/).

### Segmentation of images

**Figure 3:**
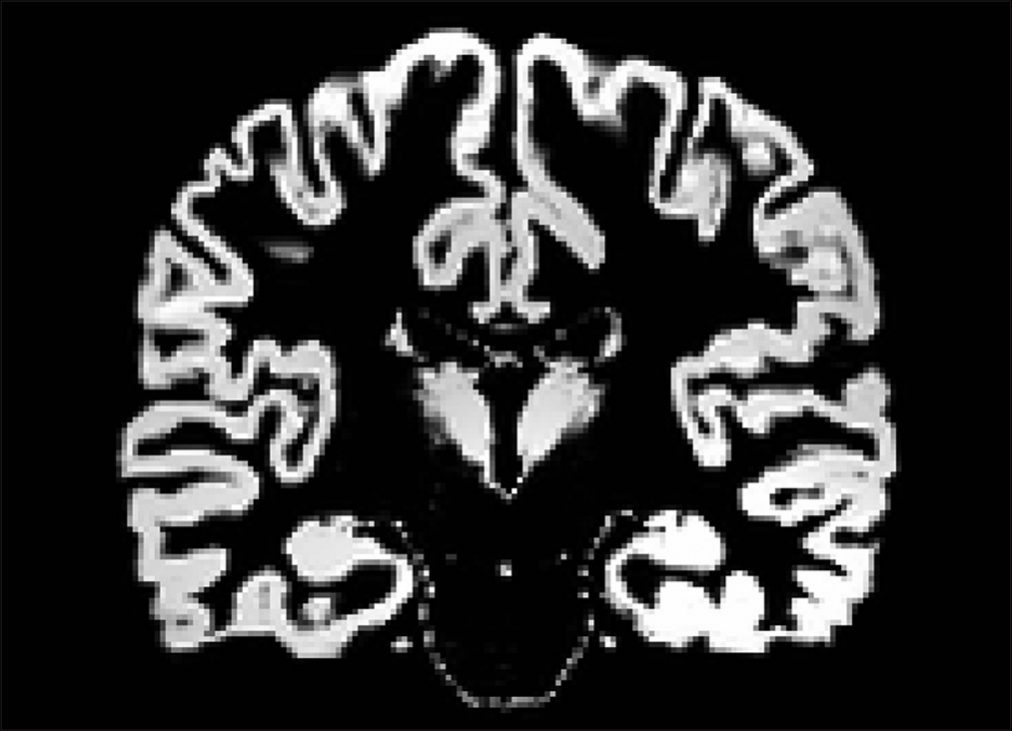
Segmented image of brain showing gray matter segmentation

Segmentation is a fully automated procedure to remove scalp tissue, skull, and dural venous sinus voxels. Segmentation of images was done with Statistical Parametric Mapping (SPM)-5 software, which employs a mixture-model cluster analysis. These series of operations yields extracted gray and white matter and cerebrospinal fluid partitions. Gray matter partitions were used for the thalamus volume measurements [Figure 3]. Intracranial volume was calculated by summation of the individual volumes of these partitions, using the methodology described previously.[36]

### Region of interest measurement

**Figure 4:**
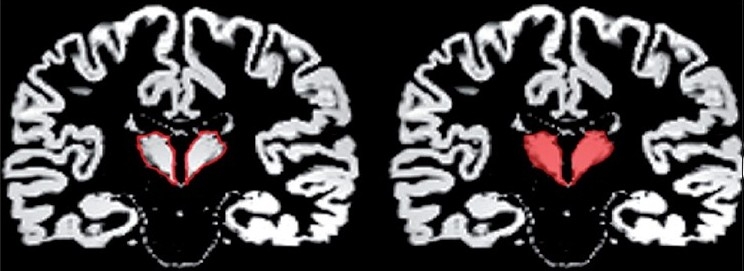
Manual tracing of the thalamus with outline in red (left) and whole volume in red (right)

Using coded images, separate measurements were obtained for the left and right thalamus by using a manual tracing technique. The measurements were carried out in the coronal sections [Figure 4]. The mamillary body was used as the anterior boundary. The internal capsule was considered the lateral boundary, the third ventricle the medial boundary, and the inferior border of the third ventricle the inferior boundary. The posterior boundary was defined by where the hemispheres of the thalamus merged under the crux fornix. The superior boundary was the main body of the lateral ventricle [Figures 3 and 4]. The thalamus was marked similarly on both left and right sides and individual volumes were computed in millilitres (ml).

### Inter-rater and intra-rater reliability and blinded rating

Two raters (NPR and SK) measured the thalamus volumes of five subjects independently, and excellent inter-rater reliability was established with an intra-class correlation coefficient of >0.9. Subsequently all ROI measurements were done on coded images by a single well-trained and reliable rater (NPR) who was blind to the subject identification and clinical data. At the end of the measurement of all images, the rater (NPR) re-measured thalamus volumes of five recoded images and a good intra-rater reliability was established, with an intra-class correlation coefficient >0.8.

### Statistical analysis

The statistical analysis was performed using the Statistical Package for Social Sciences v. 13.0. The sociodemographic and clinical data were compared using the independent samples *t*-test and the chi-square test. The effect of the status (schizophrenia patients *vs* healthy control) on the thalamic volume was examined using the analysis of covariance (ANCOVA), with intracranial volume as the covariate. Pearson correlation analysis was employed to examine the relationship between the morphometric measurements and illness-related variables. The statistical significance was set at *P*<.05 (two-tailed).

## RESULTS

### Sociodemographic and clinical variables

There was no significant difference in age, sex ratio, education, and socioeconomic-status (as measured by monthly family income) between the two groups: schizophrenia patients and controls [Table 1]. The educational status was recorded as a categorical variable as follows - [patient: controls - illiterate 3:3, primary school 1:3, middle school 7:8, high school 3:0, diploma 1:2 and graduate 3:3]. There was no significant difference in the educational status between the two groups (χ^2^=4.37, *P*=.49).

**Table 1:** Comparison of sociodemographic and clinical variables between patients and healthy controls

Variable	Patient group (n=18) (mean±S.D)	Control group (n=19) (mean±S.D)	t/χ^2^	*P*
Age (years)	29.22±5.69	26.53±7.29	1.24	0.22
Sex (M:F)	11:7	9:10	0.703	0.51
Monthly family income (in INR)	1781.2±423.0	2292.8±1522.3	1.2	0.20

The mean age at onset of psychosis was 28.1±6.0 years and the mean duration of untreated psychosis was 21.7±1.7 months. Psychopathology scores were available in all patients: the mean SAPS score was 32.5±12.7 and SANS score was 64.1±29.2.

### Region of interest–thalamus volume analysis

**Figure 5:**
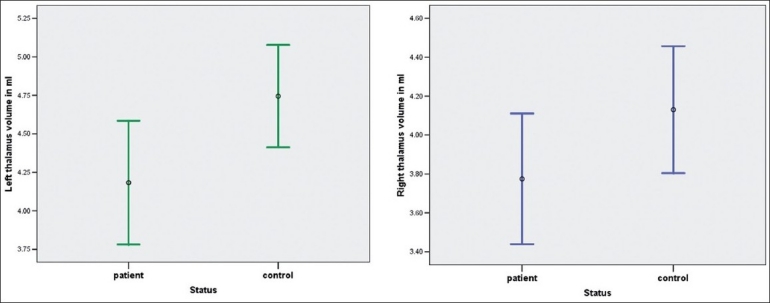
Error bars showing comparison of Right and left thalamus volumes (ml) between patient and control groups

The intracranial volume in milliliters (mean±SD) of the patients was 1300.2±150.7 and that of the controls was 1330.32±176.1. On ANCOVA using intracranial volume as a covariate, the total thalamus volume and the right thalamus and left thalamus volumes were significantly smaller in schizophrenia patients in comparison to healthy subjects [Table 2 and Figure 5].

**Table 2:** Comparison of thalamus volumes between patient and control groups

Brain volume (ml)	Patient group (n=18) (mean±S.D)	Control group (n=19) (mean±S.D)	F$	P*
Right thalamus	3.77±0.67	4.13±0.61	5.25	0.02
Left thalamus	4.18±0.80	4.74±0.69	6.98	0.01
Total thalamus	7.9±1.14	8.8±1.44	6.56	0.01

$ Analysis of covariance using intracranial volume (ICV) as covariate

* Significant at *P*<.05

### Relation between symptom dimensions and thalamic volumes

The thalamic volumes had significant contrasting correlations with symptom dimensions: significant negative correlation with SANS score and significant positive correlation with SAPS score. There was a significant negative correlation between SANS score and right thalamus volume (*r*=−0.52; *P*=.03), left thalamus volume (*r*=−0.50; *P*=.04), as well as total thalamus volume (*r*=−0.52; *P*=.03). There was a significant positive correlation between SAPS score and right thalamus volume (*r*=0.55; *P*=.02), left thalamus volume (*r*=0.53; *P*=0.03), and total thalamus volume (*r*=0.55; *P*=.02). This indicates that the smaller the thalamus volumes the more the negative symptoms, and the larger the thalamus volume the more the positive symptoms. There was no significant correlation between thalamus volumes and the age of the patient or the age at onset of psychosis.

## DISCUSSION

To the best of our knowledge, this the first study to use high-resolution 3-Tesla MRI to compare the thalamus volume in anti-psychotic-naïve schizophrenia with that in matched healthy controls. The results of this study demonstrate significantly smaller right, left, and total thalamus volumes in anti-psychotic-naïve schizophrenia patients compared to healthy controls. In patients, there was also a significant negative correlation between the thalamus volume and negative symptoms score and significant positive association between thalamus volume and positive symptoms score; that is, the smaller the thalamus the greater the negative symptoms, and the larger the thalamus the greater the positive symptoms.

Our findings are in accord with earlier studies reporting smaller thalamus volume in patients with schizophrenia. A meta-analysis and recent reviews support the view that there is a thalamus volume deficit in schizophrenia.[13,16,17] The findings of this study replicate the results of earlier studies on anti-psychotic-naïve schizophrenia using 1.5-Tesla MRI studies.[23,37,38] The association between thalamus volume and negative symptoms (i.e., the smaller the thalamus the higher the negative symptoms) is in tune with an earlier report that showed a significant correlation between the thalamic volumes and negative symptoms in first-episode patients but not in chronic patients. Importantly, the inclusion of anti-psychotic-naïve subjects and the use of high-resolution imaging in the present study eliminated the effect of potential confounding factors that was present in the earlier studies.

### Thalamus and schizophrenia

The thalamus is a vital relay station and crucial in interconnecting different cerebral structures, particularly the cerebral cortex. The thalamus has afferent and efferent connections with multiple regions in the cortex and is fundamental for ‘filtering’ or ‘gating’ sensory input to the cortex.[39] The thalamus consists of different nuclei that project to the sensory and motor cortex and receives information back from these cortical regions. In addition, it also has projections to, and receives information from, the prefrontal cortex. This function of the thalamus involves cortico-striato-thalamic loops, which in turn are modulated by the cerebral cortex.[5] Thus, in addition to the role as a sensory relay center, the thalamus is also part of frontal, limbic, and cerebellar circuits and plays an important role in the regulation of cognition, affect, and behavior. This is exemplified by the wide-ranging behavioral disturbances associated with lesions of the thalamus.[40]

Evidence from different sources suggests the involvement of a cortico-striato-thalamic loop in the pathogenesis of schizophrenia.[5,40] The subcortical structures are richly innervated by dopaminergic, glutamatergic, and serotonergic neurons, and subcortical neurotransmitter imbalance is the proposed mechanism for the pathogenesis of schizophrenia.[5] The thalamus also forms an important part of the cortico-cerebellar-thalamic-cortical circuit (CCTCC): fibers from the cortex reach the cerebellum through the pons and, in turn, cerebellar fibers are relayed into the thalamus, with thalamocortical fibers carrying the impulses back to the cerebral cortex. The CCTCC is said to be the circuitry for cognitive function and acts as the coordinator of mental processes. Disruptions in this circuit results in cognitive dysmetria, which is proposed to be the basic deficit leading to symptoms of schizophrenia.[41,42] Hence, our finding of smaller thalamus volumes in patients with schizophrenia supports the role of defective thalamus function (in turn resulting in aberrant activity of cortico-striato-thalamic circuit and CCTCC) in schizophrenia.

### Thalamus and symptom dimensions of schizophrenia

As the thalamus plays a central role in filtering or gating sensory inputs, disruption of the thalamus due to injury or some neurodevelopmental aberration can result in deficits in ‘sensory gating’ and may play an important role in symptom formation in schizophrenia.[5,6] Earlier reports have suggested that lesions in the cortico-striato-thalamic circuit may induce a ‘disinhibition syndrome’ and an increased sensitivity to amphetamine.[5] It is proposed that patients with early schizophrenia are ‘flooded by sensory impressions from all quarters,’ leading to a difficulty in distinguishing between the ‘self’ and the ‘not self’ when the central processing apparatus is not able to efficiently handle this supernormal influx of stimuli. A problem with filtering stimuli and determining ego boundaries could lead to positive symptoms, such as the experience of hearing voices or the feeling of being overwhelmed and persecuted.[42,43]

On the other hand, several studies have documented the association of negative symptoms with reduced gray matter volume in different brain regions. Left temporal lobe, anterior amygdala, anterior cingulate, and medial frontal cortex gray matter volume reductions are reported to be associated with negative symptoms.[24–27] Thus, negative symptoms in schizophrenia are associated with more tissue loss and this is consistent with Crow’s hypothesis of type 1 and type 2 schizophrenia. A few studies have examined the relation between negative symptoms and thalamic volumes; though some studies did not find a reduction in thalamus volume in comparison to healthy controls, there was significant correlation between negative symptoms and volume reduction.[13,37]

Thus, with the available data, an aberrant activity of the subcortical and cortical structures appear to be associated with positive symptoms, and a deficit in the volume of regional brain tissue is associated with negative symptoms. A neurodevelopmental model more consistently explains both the positive and negative symptoms. During the development of the human brain, reorganization of cortical connections takes place through a programmed synaptic restructuring called pruning.[44] According to the neurodevelopmental hypothesis of schizophrenia, aberrant migration or impaired pruning may compromise the thalamo-cortical circuitry and result in symptoms of schizophrenia.[1] Interestingly, neuropathological studies have reported abnormal thalamic projections to the prefrontal cortex and nongliotic neuronal loss to support the above hypothesis.[45–47]

The aberrant pruning can result in either hyperconnectivity due to failure of elimination of synapses (known as ‘pathological blooming’) and lead to positive symptoms or it may result in hypoconnectivity due to ‘pathological excessive pruning’ and lead to negative symptoms.[28] Thus, neurodevelopmentally, contrasting pruning aberrations can result in either the positive or the negative symptoms of schizophrenia. Therefore one would expect the positive symptoms of schizophrenia to be associated with aberrant activity of the thalamus due to pathological blooming (reflected by a relatively larger thalamus volume) and negative symptoms to be associated with an excessive pathological pruning (resulting in volume deficits). Our finding of contrasting correlation of thalamic volume with positive and negative symptoms supports the above hypothesis.

### Methodological issues

Our study has several methodological strengths: 1) To the best of our knowledge, this is the first study using a high-resolution 3-Tesla MRI scan in anti-psychotic-naïve schizophrenia patients. High-resolution improved slice selection and measurement accuracy. Also, importantly, examining anti-psychotic-naïve patients eliminated the potential confounding effects of neuroleptic treatment. 2) Volumetric assessments was done using ROI-based manual morphometry with coded images and with the rater being blinded to the status. 3) Good intra-rater and inter-rater reliability was established. 4) We used objective established criteria for defining the boundaries of the thalamus. 5) Psychopathology was comprehensively assessed using SAPS and SANS, with good inter-rater reliability. 6) The diagnosis was reliably ascertained by independent assessments by two qualified psychiatrists using MINI-Plus and clinical interview. And, finally, 7) we used healthy controls group-matched for age, sex, handedness, education, and socioeconomic status. These rigorous methodological factors add further value to our study observations.

## CONCLUSIONS

In summary, our study demonstrated that anti-psychotic-naïve schizophrenia patients have significantly smaller right thalamus, left thalamus, and total thalamus volumes as compared to matched healthy controls. In addition, there were significant contrasting correlations between thalamic volumes and psychopathology dimensions suggestive of contrasting pruning aberrations. Together, these findings suggest a neurodevelopmental pathogenesis in schizophrenia.
